# Multicenter Study on Communication, Language and Speech in Italian Children with Cerebral Palsy—Survey, Assessement Protocols and Proposal for a Classification System

**DOI:** 10.3390/children13050586

**Published:** 2026-04-23

**Authors:** Elisa Granocchio, Claudia Maggiulli, Luca Andreoli, Stefania Gazzola, Ilaria Pedrinelli, Santina Magazù, Daniela Sarti, Marinella De Salvatore, Martina Paini, Sara Rinaldi, Sara Visentin, Anna Salvalaggio, Sara Scotto, Elisabetta Cane, Elvira Bargagni, Elena Giordano, Sabrina Signorini, Miriam Corradini, Ivana Olivieri, Ilaria De Giorgi, Maria Carmela Oliva, Antonio Trabacca, Elisa Fazzi, Serena Micheletti, Cristina Marinaccio, Elena Grosso, Emanuela Pagliano

**Affiliations:** 1 Department of Pediatric Neuroscience, Fondazione IRCCS Istituto Neurologico “Carlo Besta”, 20133 Milan, Italy; elisa.granocchio@istituto-besta.it (E.G.); stefania.gazzola@istituto-besta.it (S.G.); ilaria.pedrinelli@istituto-besta.it (I.P.); santina.magazu@istituto-besta.it (S.M.); daniela.sarti@istituto-besta.it (D.S.); marinella.desalvatore@istituto-besta.it (M.D.S.); martina.paini@istituto-besta.it (M.P.); emanuela.pagliano@istituto-besta.it (E.P.); 2 Department of Education Sciences, University of Genoa, 16128 Genoa, Italy; 3 Department of Humanities and Life Sciences, University School for Advanced Studies, Istituto Universitario di Studi Superiori—IUSS, 27100 Pavia, Italy; 4 AULSS 6 Euganea, UOC Infanzia Adolescenza Famiglia e Consultori, 35131 Padova, Italy; sara.rinaldi@aulss6.veneto.it (S.R.); sara.visentin@aulss6.veneto.it (S.V.); 5 Neuropsichiatria Infantile CDR, 10124 Torino, Italy; anna.salvalaggio@aslcittaditorino.it (A.S.); sara.scotto@aslcittaditorino.it (S.S.); elisabetta.cane@aslcittaditorino.it (E.C.); 6 Azienda Usl Toscana Centro, Gruppo Attività Interdisciplinare Disordini Infantili Neuromotori, 50135 Firenze, Italy; elvira.bargagni@uslcentro.toscana.it (E.B.); elena.giordano@uslcentro.toscana.it (E.G.); 7 Fondazione IRCCS Mondino, 27100 Pavia, Italy; sabrina.signorini@mondino.it (S.S.); miriam.corradini@mondino.it (M.C.); 8 Centro Benedetta D’Intino, 20156 Milan, Italy; ivana.olivieri@benedettadintino.it; 9 Fondazione IRCCS Don Carlo Gnocchi, 20148 Milan, Italy; idegiorgi@dongnocchi.it; 10 La Nostra Famiglia Association IRCCS “E. Medea” Scientific Hospital for Neurorehabilitation, 72100 Brindisi, Italy; mariacarmela.oliva@lanostrafamiglia.it; 11 Scientific Institute IRCCS “E. Medea”, Scientific Direction, 23849 Bosisio Parini, Italy; antonio.trabacca@lanostrafamiglia.it; 12 Unit of Child Neurology and Psychiatry, ASST Spedali Civili of Brescia, 25123 Brescia, Italy; elisa.fazzi@unibs.it (E.F.); serena.micheletti@unibs.it (S.M.); 13 Ospedale Regina Margherita, 10126 Torino, Italy; cristina.marinaccio@unito.it (C.M.); elena.grosso@unito.it (E.G.)

**Keywords:** cerebral palsy, speech and language disorders, child neurology, early detection and intervention, augmentative and alternative communication

## Abstract

**Highlights:**

**What are the main findings?**

- Standardized protocol development: The ‘Italian CP & Language Network’ developed three age-specific assessment protocols (early language, school-age, and minimally verbal) to address the high variability in clinical practices for children with cerebral palsy.
- Unified classification system: A new multi-level classification system for speech and language disorders was established to ensure diagnostic consistency and identify specific needs for augmentative and alternative communication (AAC).

**What are the implications of the main findings?**

- Enhanced clinical precision: Standardizing these assessments allows for earlier identification of communicative vulnerabilities, enabling more timely and tailored therapeutic interventions across different developmental stages.
- Multicenter research foundation: This framework provides a standardized basis for future multicenter studies to correlate linguistic phenotypes with neuroradiological data and motor outcomes in the CP population.

**Abstract:**

Background: Communication, language, and speech disorders are highly prevalent in children with cerebral palsy (CP) and substantially impact social, educational, and community participation. However, few studies have systematically characterized communicative and linguistic profiles using standardized assessments. This paper outlines the work of the ‘Italian CP & Language Network’ over the last two years, focusing on identifying research priorities, developing specialized assessment protocols, and proposing a shared classification system for speech and language disorders in children with CP. Methods: A survey was sent to 11 specialized centers to investigate clinical practices and assessment tools. Based on the results and an extensive literature review, the group developed three age- and complexity-based diagnostic protocols and a shared classification system. Results: The survey highlighted high variability in test selection, especially for speech and pragmatic assessment, and a significant need for ad hoc tools for augmentative and alternative communication (AAC). Three standardized protocols were defined: (1) early language (<48 months), (2) school-age language and pragmatics (4–12 years), and (3) minimally verbal children (6–12 years). A multi-level classification system for language and speech disorders was proposed to improve diagnostic consistency. Conclusions: Standardizing assessment is a critical step toward early identification of communicative vulnerabilities to guide tailored interventions and promote participation and quality of life across developmental stages. The group provides a framework for prospective multicenter data collection to correlate linguistic and speech phenotypes with neuroradiological features and motor outcomes.

## 1. Introduction

Cerebral palsy (CP) is one of the most common causes of childhood disability, with a current overall birth prevalence (including post-neonatal) of 1.6 per 1000 live births [1]. It is defined as an early-onset, lifelong neurodevelopmental condition characterized primarily by impairments in movement and posture, but it is also frequently associated with sensory, cognitive–neuropsychological, emotional–relational, and communicative difficulties [2]: “The classification of CP subtypes based on clinical features identifies three main groups: spastic, dyskinetic, and ataxic cerebral palsy. To describe involvement of both sides and one side of the body, bilateral and unilateral forms are respectively used [3].”

Communication disorders are particularly frequent in this population, with prevalence estimates ranging from 42% to 74% in case-based studies [4,5,6,7]. These disorders manifest as a reduced ability to send and/or receive both verbal and non-verbal messages, resulting in reduced social, educational, and community participation [5,8]. Children with CP and communication impairments consistently report lower quality of life compared with their peers with CP who do not have such difficulties [9,10], underscoring the clinical and rehabilitative importance of early identification and intervention.

Communication challenges in CP arise from a combination of verbal and non-verbal factors. Non-verbal difficulties may include limited eye contact, restricted facial expression, or reduced use of gestures. Verbal communication may be impaired in two distinct ways. Firstly, speech difficulties may be encountered, i.e., the child may experience an inability to co-articulate the sounds of language. Secondly, linguistic difficulties may be present, i.e., the child may struggle to produce or comprehend words and sentences. Moreover, children with CP frequently face challenges in acquiring literacy skills, which further restricts access to written language. Beyond these aspects, communicative abilities in children with CP are also affected by cognitive and perceptual/sensory deficits, as well as by the interaction of these multiple contributing factors [8].

A review of the literature indicates that most children with CP do indeed develop verbal language, albeit with occasional delays and challenges. However, it is noteworthy that approximately one-quarter of these children present with severe dysarthria/anarthria, necessitating alternative means of communication [7,11,12,13]. Receptive language skills are also common but remain understudied, especially among minimally/non-verbal children, partly because of a lack of appropriate assessment tools [14,15].

To date, reported prevalence rates for expressive and receptive language disorders range from 37% to 86% [7,12,13], while speech disorders are estimated to affect more than 70% of children with CP [13,16].

A significant number of studies have focused on the search for early predictors of later linguistic, articulatory, and cognitive outcomes. Overall, greater communication disabilities have been observed in children with dyskinetic CP, followed by those with bilateral spasticity, whereas children with unilateral spasticity generally have the most favorable prognosis [17,18,19]. Greater impairment in verbal comprehension has been associated with both poorer gross motor function (GSMF IV–V) and reduced manual skills. Across CP subtypes, spastic forms have been linked to a higher prevalence of verbal comprehension difficulties compared with dyskinetic forms [19,20]. Finally, cognitive functioning has been found to be strongly correlated with verbal comprehension, with poorer receptive skills observed in children with CP and co-occurring disorders of intellectual development [4,17,18,19].

Recent evidence suggests that early articulatory markers may predict later speech outcomes. Children who produce fewer than six phonemes at 24 months or who display delayed, monotonous babbling that fails to progress to more complex syllabic structures are at elevated risk of developing dysarthria [21]. Comorbid epilepsy, visual impairments, or feeding difficulties are additional factors for poorer language outcomes [22].

From a methodological perspective, numerous studies rely only on data from disease registries when assessing children’s verbal language acquisition [5,7], while others have utilized indirect assessments of communication skills through questionnaires, such as the Vineland Adaptive Behavior Scales (VABS) [17], and checklists, including the Communication and Social Behavior Scales Developmental Profile (CSBS-DP) [23,24]. The majority of studies have primarily utilized standardized systems to classify communication functioning, such as the Communication Function Classification System (CFCS) [18,25], the Functional Communication Classification System (FCCS) [18,26], and the Viking Speech Scale (VSS) [27], in order to categorize severity across five levels. In U.S. research, the Speech Language Profile Group (SLPG) has proposed a classification of children with CP into four distinct subgroups in accordance with the presence or absence of speech and receptive language disorders [28]. Despite these advances, standardized direct speech–language assessment remains challenging in this population [8], and most studies involve small, heterogeneous samples of children representing diverse CP subtypes [12,13,16,20,29,30].

Despite the substantial clinical pertinence and rehabilitation ramifications, there is a lack of studies reporting on the examination of linguistic and communicative competencies in children with CP, through a combination of both indirect and direct assessment instruments. Recognizing the clinical importance of shared data and standardized protocols, an Italian multicenter working group was established in 2022 within the Gruppo Italiano Paralisi Cerebrali Infantili (GIPCI). This “Italian CP & Language Network” aiming to investigate communication, language, and speech in children with CP through collaborative research, development of diagnostic protocols, and the creation of a shared classification system.

The network is currently conducting a systematic multicenter collection of both cross-sectional and longitudinal data on communication, language, and speech skills in children with CP. Correlational analysis will also investigate relationships between these clinical speech and language data and neurological, motor, and neuropsychological measures. At present, 11 Italian centers participate, involving multidisciplinary teams that include neuropsychiatrists, psychologists, speech therapists, and neuro- and psychomotor therapists of developmental age (see Supplementary S1 for the list of participating centers).

This paper reports on the work carried out over the past two years by the “Italian CP & Language Network”, with three primary aims: (1) collect data about instruments used in Italy for the assessment of communication disorders in children with CP through a survey; (2) to construct and share ad hoc communication, speech, and language assessment protocols; and (3) to propose a shared classification system for language and speech disorders in children with CP.

## 2. Materials and Methods

A multidisciplinary working group from the Fondazione IRCCS Istituto Neurologico Carlo Besta (20133, Milan, Italy), comprising experts in cerebral palsy (CP) and language disorders, developed a survey to investigate the clinical organization, patient demographics, and assessment tools used for communication and speech–language evaluations across Italian specialized centers. The questionnaires were addressed to the clinical leads of the components of the “Italian CP & Language Network” group, who had to collect all the information from the whole team.

The survey was distributed via email in May 2022 to centers affiliated with the GIPCI network (Gruppo Italiano Paralisi Cerebrali Infantili). The questionnaire was structured with multiple-choice questions to explore several key domains: multidisciplinary staff composition; frequency of language assessments; and functional profiles of patients based on the Gross Motor Function Classification System (GMFCS) [31]. We also collected data on standardized and non-standardized tools for communication, speech, and language assessment, clinical experience with augmentative and alternative communication (AAC), assessment protocols for cognitive development and visual–perceptual skills, and availability for research collaboration in relation to children’s characteristics. Responses were collected using Google Forms (URL: https://docs.google.com/forms/u/0/, accessed on 10 March 2026). Data were then exported to Microsoft Excel (2013) for extraction and descriptive statistical analysis.

From survey results, three protocols were defined based on the children’s age and the complexity of CP. These include the Italian standardized assessment of communication, speech, and language, with the lexicon and syntax domains divided into comprehension and production.

Concurrently, the research group conducted a comprehensive literature review to disseminate the most recent scientific evidence about speech and language disorder classification systems to foster the adoption of a shared framework. This was discussed during monthly online meetings, involving all the working group members.

These assessment protocols were approved by the Institute’s Ethics Committee in February 2023 (CET14/23) and subsequently amended in April 2024 by the Territorial Ethics Committee (CET15/24).

**Figure 1:**
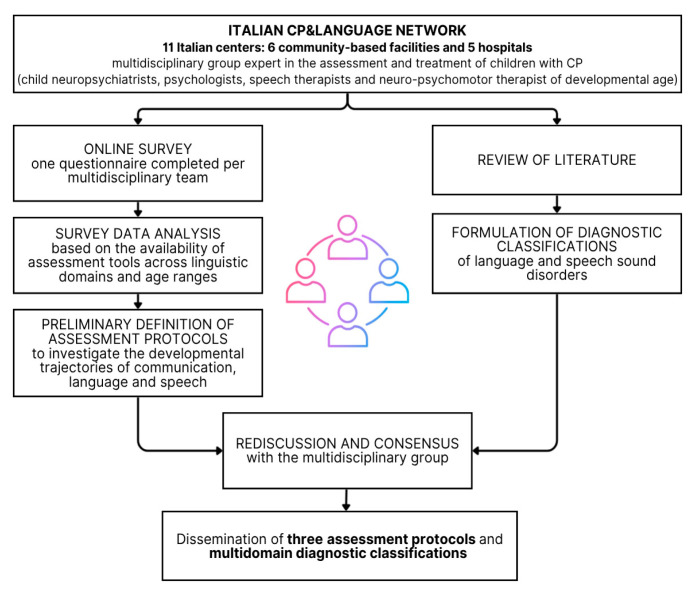
Flowchart illustrating the development of the protocols and the conditions/criteria for decision-making protocols and multidomain diagnostic classification.

Figure 1 illustrates the process of this study.

## 3. Results

### 3.1. Survey

Eleven Italian centers from the GIPCI network participated in the survey. All centers provide assessment and treatment for children with CP; six are community-based facilities, while the others are hospitals. Geographically, nine centers are located in Northern Italy, one in Central Italy, and one in Southern Italy. Regarding professional roles, child neuropsychiatrists are present in 100% of the centers. Speech and language therapists (SLTs) and neuro- and psychomotor therapists of developmental age are represented in 90% of the facilities. Physiotherapists are present in 70%, while occupational therapists are available in 30% of the surveyed centers. The survey revealed that 80% of the centers primarily treat children with GMFCS levels I–III, while 50% reported seeing patients with higher severity (GMFCS IV–V). Experience with patients requiring AAC was reported by 63% of the centers. There is high variability in the choice of assessment tools for speech and language assessment. While there is a preference for standardized tests, this trend is less consistent for speech evaluation (e.g., intelligibility and articulatory variability), where clinical judgment is more frequently adopted. The use of specific standardized tests for AAC remains limited. About cognitive assessment, for preschoolers, the Griffiths III (72.7%) [32] and WPPSI III/IV (81.8%) [33,34] are the most utilized tools, while the Bayley scales [35] are used by only 9.1%. For school-age children, the WISC-IV (81.8%) [36] is the primary tool. For children with significant functional or motor impairment, centers reported using the Leiter-III (27.3%) [37] and Raven’s Colored Progressive Matrices (9.1%) [38]. The Test of Visual Perception—TPV [39] and the of Visual–Motor Integration—VMI [40] are the most common tools, both used by 72.7% of centers. Only 54.5% of the centers have direct access to MRI neuroimaging, while the remainder rely on written reports. A strong interest in collaborative research was identified: 90.9% of centers expressed interest in studying minimally verbal children, 63.6% in children aged 5–7 years, and 36.4% in early assessment for children under 36 months. Assessment tools for communication, language, speech, and AAC used across clinical centers are reported in Table 1.

### 3.2. Protocols

Based on survey results, discussion between working group members, and the literature review, three assessment protocols were developed to investigate the developmental trajectories of communication, language, and speech. The justification for our selection lies in the tools’ proven reliability and standardized nature. These instruments were chosen because they are widely utilized in specialized centers, allow for longitudinal assessment, and have already demonstrated validity across various populations, making them highly suitable for the target CP group. In particular, these are designed to better characterize communicative, linguistic, and speech phenotypes. Here are the specifics of each protocol: (1) early language and communication prerequisites in children with CP younger than 48 months; (2) school language, speech, and pragmatics in children with CP aged 4–12 years; and (3) AAC evaluation in children with CP aged 6–12 years who are minimally verbal or non-verbal and could therefore benefit from alternative and augmentative means of communication.

The decision to assess minimally verbal children after the age of six was supported by both clinical observation and the literature, which indicates that in more severe cases, language development may occur later than expected. The proposed protocols follow a hierarchical structure, allowing clinicians to estimate the child’s linguistic level: when children score below the range for the age-appropriate test, clinicians are encouraged to use a test for younger children to assign a linguistic age equivalent. This is in order to better describe the skills acquired and to derive a language quotient, indexing the language development relative to chronological age.

As a preliminary step before protocol administration, children with CP should undergo a neurological examination to assign a level based on shared international classifications [65]: in particular, children are classified according to: the Gross Motor Function Classification System-Expanded & Revised—GMFCS-E&R [31] and the Manual Ability Classification System—MACS [66] (or Mini-MACS for children from 1 to 4 years) for motor function; the Communication Function Classification System—CFCS [25] and the Eating and Drinking Ability Classification System—EDACS (or Mini-EDACS for children from 18 to 36 months) [67] for communicative and feeding abilities; and the Visual Function Classification System—VFCS [68] for visual skills.

Each assessment protocol contains a comprehensive, standardized test battery investigating the following key areas:

- Neuropsychological skills assessed through age-appropriate tests of psychomotor development or intelligence (Griffiths III [32] or Wechsler Scales of Intelligence: WPPSI III or IV [33,34], WISC IV or V [36,69]). When standardized testing is not feasible, adaptive functioning should be evaluated using caregiver interviews (Vineland Adaptive Behavior Scales/VABS or Adaptive Behavior Assessment Test/ABAS-II [70,71]), complemented by non-verbal assessment (Leither III, Raven-CPM [37,38]) and clinical observation;
- Visuo-perceptive abilities through the Test of Visual Perception—TPV or test of Visual–Motor Integration—VMI [39,40];
- Communication and language assessment by standardized tests or parent report measures of receptive and expressive lexical and grammatical skills, and communicative participation (see S2);
- Oral–motor and speech assessment, including oral structure integrity, articulatory accuracy, co-articulation, diadochokinesis, pneumophonic coordination, vocal quality, lexical variability, and speech intelligibility (see S2);
- Augmentative and alternative communication: an ad hoc caregiver interview was developed for minimally verbal or non-verbal children (see S2).

The complete communication, speech, and language protocols are available as a Supplementary Material (see S2).

### 3.3. Diagnostic Classifications

Following a comprehensive review of prevailing terminology and classifications of language disorders—including the Diagnostic and Statistical Manual of Mental Disorders, Fifth Edition, Text Revision [72], the Delphi consensus study on language impairments in children [73], and the Italian Consensus Conference [74]—the group proposes the term “language disorder” to denote language impairments arising from neurological conditions. The distinction between “delay” (Expressive Delay/Receptive Delay) and “disorder” (Expressive Disorder/Receptive Disorder) was considered clinically meaningful in order to differentiate children who develop language late but follow a typical trajectory from those who exhibit atypical developmental pathways associated with a more severe prognosis [75] (see Table 2). For a more comprehensive description, we also decided to specify whether children presented with associated difficulties in pragmatic–communicative skills. Similarly, based on a comprehensive review of current classifications of speech sound disorders (SSDs) [76,77,78,79], together with alternative frameworks specifically addressing SSDs in children with CP [16], the group formulated a classification proposal. Motor speech disorders (MSDs) have been differentiated into three primary diagnostic categories according to Shriberg [76]: Motor Speech Delay (MSD), dysarthria (DYS), and Childhood Apraxia of Speech (CAS). To describe certain speech features that are often present in children with CP but are not considered core features of CAS or DYS, additional diagnostic categories have been introduced: Phonetic delay/disorder to describe a speech–motor-based problem in the production of phonemes (execution of articulatory gestures) and Phonological delay/disorder to describe the presence of typical or atypical speech sound errors, which can be secondary to either speech–motor or perceptual–linguistic causes. The diagnosis of CAS was established according to the three core diagnostic characteristics outlined by the American Speech–Language–Hearing Association [80], with the support of the checklist proposed by Iuzzini-Seigel [81]. Moreover, the diagnosis of DYS was established according to the support of a perceptual rating of dysarthria (modified version of the Mayo Clinic dysarthria classification system [16]) (see Table 2B). According to this proposal, a child could receive multiple diagnoses based on their specific speech characteristics (e.g., DYS due to poor phonatory support and impaired voice quality, combined with a phonetic disorder due to a restricted phonetic inventory). For a more comprehensive description, we also decided to specify whether children presented with associated difficulties in oral praxis, feeding, and/or swallowing.

This approach to classifying speech and language allows us to place children along a developmental continuum, ranging from typical development to mild impairments in certain skills (delays)—which can still have a functional impact—to more severe impairments in other areas (disorders).

This framework also adopts a multi-level descriptive approach, allowing for multiple co-occurring diagnoses rather than a single one. Assigning more than one diagnosis enables a comprehensive definition of each child’s profile across the assessed domains.

In Table 2A, proposed classifications of speech and language disorders are presented. In Table 2B, proposed classifications of SSDs in children with CP are presented.

## 4. Discussion

Children with CP represent a particularly complex clinical population due to their marked phenotypic heterogeneity, encompassing a broad spectrum of motor, cognitive, and sensory impairments. Consequently, communication assessment in this population is especially challenging. Early identification of communication and language difficulties is essential to ensure timely referral of children with CP to appropriate intervention pathways, which is consistent with current guidelines for infants under 24 months who are at high risk of CP [82].

In this paper, we describe the methodological and collaborative effort undertaken by the Italian CP & Language Network to define shared assessment protocols and classification systems. Inspired by the model of Surveillance of Cerebral Palsy in Europe (SCPE), which provides standardized definitions and data-collection procedures for CP epidemiology [1,2,3], this initiative aims to harmonize clinical practice in the assessment of communication disorders—an area that is not systematically addressed within existing epidemiological registries.

The primary objective of this initiative was to establish the foundations for prospective multicenter data collection across the Italian territory.

Recent surveys conducted in other countries, including the United States and Germany, have highlighted a general lack of confidence among SLTs in identifying and classifying motor speech disorders—particularly dysarthria—and a limited use of specific assessment approaches [82,83].

Our survey confirms similar trends among Italian specialists. In the evaluation of speech, communicative participation, and pragmatic aspects, the use of non-standardized assessments and clinical observation emerged as the most prevalent practices. Based on these findings, the most common tests were selected and integrated with lesser-known standardized instruments to assess speech and communicative participation. Regarding receptive and expressive language, the two most widely utilized instruments are the MacArthur–Bates Communicative Development Inventories (CDI)—specifically the Italian version (PVB) [41]—and the BVL battery [45].

It is noteworthy that no single instrument currently available covers both the initial and subsequent stages of language development. Furthermore, a lack of homogeneity in test selection was observed for the 36-to-48-month age range. Similarly, assessment tools for language comprehension in children under 42 months appear to be underutilized.

During a series of online meetings, lesser-known tools were presented to ensure proficiency across all participating centers and to facilitate the sharing of specialized assessment protocols.

The evaluation of pragmatic competence remains a significant challenge, mirroring difficulties encountered in other clinical populations. Only a few AAC instruments are currently in use within clinical services; this highlights the urgent need to develop ad hoc tools to better assess minimally verbal children.

Through the survey, we identified the assessment tools most commonly used across centers and reached consensus on their adapted use. It was also agreed that, for children with more severe CP, assessment tools designed for younger age groups could be administered to determine developmental language age. Given the hierarchical structure of many standardized protocols, this approach allows for the derivation of language quotients.

Specific instruments—particularly those related to speech assessment and communicative participation, which were not universally known—were presented to ensure broader dissemination across working groups. Discussion within the working groups also highlighted the need to develop an ad hoc, rapid, and user-friendly tool for assessing the use of AAC. A dedicated task force was therefore established, resulting in the development of a new instrument that is currently under review for publication.

From these observations, three protocols that were age- and complexity-based were defined. The primary aim was to delineate the communicative–linguistic profile of each individual child and to initiate systematic data collection of homogeneous data, which represents the only viable strategy for clinicians to better understand this population. Standardized assessments provide a baseline against which individual progress can be monitored over time and enable evaluation of intervention effectiveness [84]. However, clinical discussion and the limited available literature confirm the difficulties inherent in assessing these children—not only because of their clinical characteristics but also due to the lack of ad hoc assessment tools (e.g., tools accounting for sensory and motor impairments). In addition, the presence of co-occurring neurodevelopmental disorders, such as attention-deficit/hyperactivity disorder and autism spectrum disorder—which are highly prevalent in children with CP [85,86]—may further influence task engagement and performance. As a result, evaluations are often conducted using qualitative analyses or schedules developed independently by SLTs. When standardized tests are employed, they are frequently adapted (e.g., enlarging pictures, modifying response sheets to allow children to point to answers, or permitting longer response times) [8,84]. Although such adaptations are frequently necessary in clinical practice, the literature highlights that the use of standardized instruments in populations with complex motor impairments requires careful interpretation and methodological transparency [8]. Within this framework, the shared tools represent an initial step toward the harmonization of assessment practices across centers while maintaining the flexibility required when working with children presenting severe motor and sensory impairments.

Clinical evaluation remains central to the assessment of language and speech sound disorders, as objective measures are not yet widely available. These may be derived in the future from the broader—though not yet imminent—use of tools such as acoustic analysis, electropalatography, and ultrasound, which are strongly recommended by experts in motor speech control [87].

The proposed classifications were developed in response to the need for clearer and more consistent terminology when describing language and speech disorders in children with CP.

Within this framework, the distinction between delay and disorder was retained as clinically meaningful. This differentiation allows clinicians to distinguish between children who follow a delayed yet qualitatively typical developmental trajectory and those who present atypical developmental patterns associated with a less favorable prognosis. In the context of CP, where developmental profiles are highly heterogeneous, this distinction may support more accurate clinical characterization and guide expectations regarding developmental outcomes.

Similarly, the proposed classification of speech disorders builds on existing models for children with CP [16]. The adoption of a multi-level descriptive framework allowing for multiple co-occurring diagnoses reflects the complex interaction between motor speech impairment, phonological development, and language in this population. In line with recent perspectives in the scientific literature [87,88], our diagnostic approach can be considered multidomain; furthermore, an effort is made to describe each child across communication and oral function, while also taking into account other areas such as cognitive and motor functioning.

## 5. Conclusions and Future Prospects

Despite the central role of communication, language, and speech in the autonomy and quality of life of children with CP, the available literature remains limited, particularly in terms of shared multicenter data and standardized diagnostic protocols.

Within the collaborative framework provided by the Italian CP & Language Network, we have collected data from 11 Italian centers through an online survey, focusing on the availability of assessment tools across linguistic domains and developmental ages. Based on these data, three assessment protocols were constructed for the evaluation of communication, speech, and language in children with CP. Moreover, a shared classification system was proposed on the basis of the most recent evidence from the literature. Following more discussion and consensus within the Italian CP & Language Network, these protocols and classifications will be disseminated to improve the comparability of clinical data across centers.

Future perspectives include the collection of multicenter data using shared protocols encompassing different communicative profiles, including school-age children with CP who use verbal language, as well as children and adolescents who are minimally verbal or rely on AAC. Moreover, data will be collected from the earliest stages of language development, starting from 24 months of age, with subsequent longitudinal follow-up.

Beyond providing a comprehensive description of communication profiles in this population, such datasets will enable the exploration of associations between language outcomes, clinical characteristics, and neuroradiological findings. The identification of early clinical markers in young children with CP may further contribute to anticipating developmental trajectories and supporting earlier and more targeted interventions. Overall, the development of coordinated multicenter initiatives represents an important step toward improving clinical practice, informing intervention planning, and ultimately enhancing the participation and quality of life of children with CP.

## Figures and Tables

**Table 1:** Assessment tools for communication, language, speech, and AAC used across clinical centers.

Area	Domain	Assessment Tools/Tests	% of the Centers That Use the Tools
**Lexicon**	Comprehension	MacArthur–Bates Communicative Development Inventories—the Italian version Primo Vocabolario del Bambino—PVB [41]	90.91
		Parole in Gioco-PinG [42]	81.82
		Test Primo Linguaggio—TPL [43]	54.55
		Test Fono-Lessicale—TFL [44]	45.45
		Batteria di Valutazione del Linguaggio—BVL Comprensione Lessicale in età prescolare [45]	81.82
		Test Neuropsicologico Lessicale—TNL [46]	9.09
		Test di vocabolario percettivo Peabody—PPVT [47]	9.09
	Production	PVB [41]	81.82
		BVL—Denominazione e Articolazione e Denominazione [45]	90.91
		Test Fono-Lessicale—TFL [44]	54.55
		TPL [43]	45.45
		Test Neuropsicologico Lessicale—TNL [46]	27.27
		PinG [42]	9.09
		Test di valutazione del linguaggio—TVL [48]	9.09
		Boston Naming Test [49]	9.09
**Syntax**	Comprehension	PVB [41]	45.45
		COVER [50]	27.27
		Prove di Comprensione Grammaticale con Oggetti—PCGO [51]	18.18
		Prove di Valutazione della Comprensione Linguistica—PVCL [52]	72.73
		BVL—Comprensione Grammaticale [45]	90.91
		Test di comprensione grammaticale del Bambino—TCGB [53]	18.18
		TROG-2 [54]	18.18
		TPL [43]	18.18
	Production	PVB [41]	81.82
		TPL [44]	54.55
		BVL—Ripetizione di frasi in età prescolare e in età scolare [45]	72.73
		Ripetizione di frasi [55]	45.45
**Speech**	Articulation	BVL—Denominazione e Articolazione [45]	90.91
		Prove di articolazione su elicitazione Fanzago [56] o Rossi [57]	27.27
	Intelligibility	Non-standardized assessments/clinical observation	72.73
		ICS-I [58]	9.09
	Diadocokinesis	Non-standardized assessments/clinical observation	63.64
		Robbins e Klee italian adaptation [59]	9.09
	Lexical variability	Non-standardized assessments/clinical observation	81.82
		Robbins e Klee italian adaptation [59]	18.18
**Pragmatics**		BVL [45]	18.18
		Questionario FOCUS-I [60]	27.27
		Abilità Socio Conversazionali del Bambino—ASCB [61]	18.18
		Abilità Pragmatiche linguistiche—APL-Medea [62]	36.36
		Non-standardized assessments/clinical observation	36.36
**AAC**		VCAA [63]	36.36
		ComFor [64]	18.18
		Non-standardized assessments/clinical observation	9.09

Note: bold is used to indentify main domains, which can contain subdomains (non-bold).

**Table 2:** (**A**) Proposed classification of language disorders in children with cerebral palsy (CP). (**B**) Proposed classification of speech sound disorders (SSDs) in children with CP. SD = Standard Deviation.

(**A**)	
**Subgroup**	**Core Features**
(1) **RECEPTIVE LANGUAGE**	
(a) **Typical receptive language development**	Standardized test scores for grammatical comprehension ≥ −1.5 SD/10th percentile.
(b) **Receptive delay**	Standardized test scores for grammatical comprehension between −1.5 SD and −2 SD/5–10th percentile.
(c) **Receptive disorder**	Standardized test scores for grammatical comprehension < −2 SD/5th percentile.
(2) **EXPRESSIVE LANGUAGE**	
(a) **Typical expressive language development**	Standardized naming test scores ≥ −1.5 SD/10th percentile and morphosyntactic skills in line with age.
(b) **Expressive delay**	Standardized naming test scores between −1.5 SD/10th percentile and −2 SD/5th percentile, with morphosyntactic skills delayed for age.
(c) **Expressive disorder**	Standardized naming test scores < −2 SD/5th percentile and morphosyntactic skills considered inappropriate for any age group, not following typical developmental trajectory.
Please, indicate presence of: Pragmatic difficulties	
(**B**)	
**Subgroup**	**Criteria**
**Typical development**	No speech errors, or errors appropriate for developmental age (produced by 10% of same-age peers).
**Phonetic delay**	Phonetic inventory delayed for age, but following the typical developmental trajectory (as in a younger child).
**Phonetic disorder**	Phonetic inventory significantly deficient for age (e.g., <8 phonemes at 30 months, <12 at 36 months, <16 at 42 months, and <19 at 48 months), with the exclusion of vowels; alternatively, production of atypical phonemes for the developmental trajectory.
**Phonological delay**	Phonological processes appropriate for a younger age (primitive developmental processes).
**Phonological disorder**	Significant presence of phonological processes inappropriate for any age group (e.g., contrasting, variable, idiosyncratic, and unusual processes; systematic preference for a sound).
**Motor speech delay (MSD)**	Delay in speech precision and stability, prosody, and voice, not meeting criteria for dysarthria or CAS.
**Dysarthria**	Impairment in one or more motor subsystems of speech (articulation, respiration, phonation, resonance, and prosody) impacting speech intelligibility [16].
**Childhood Apraxia of Speech (CAS)**	(1) inconsistent production of the same word, (2) prolonged or interrupted co-articulatory transitions, and (3) dysprosody [80,81].
Please indicate presence of: Oral praxis difficulties, feeding, and/or swallowing difficulties.	

Note: Bold text is used to highlight diagnostic labels and differentiate them from core features (on the right).

## Data Availability

The original contributions presented in this study are included in the article/Supplementary Materials. Further inquiries can be directed to the corresponding authors.

## Supplementary Materials

The following supporting information can be downloaded at: https://www.mdpi.com/article/10.3390/children13050586/s1, S1: survey questionnaire and results; Survey questionnaire; S2: protocols; Table S1.1: Protocol for children < 4 years; Table S1.2: Protocol for verbal children 4–12 years; Table S1.3: Protocol for minimally verbal or non-verbal children 6–12 years [89].

## Abbreviations

The following abbreviations are used in this manuscript:

AAC	Augmentative and alternative communication
CP	Cerebral palsy
SLT	Speech and language therapist
SSD	Speech sound disorder
