# Supplementary material for: Multicenter Study on Communication, Language and Speech in Italian Children with Cerebral Palsy—Survey, Assessement Protocols and Proposal for a Classification System
Source: Children (Basel). 2026 Apr 23;13(5):586. doi: 10.3390/children13050586 (PMC13204128; doi:10.3390/children13050586)
Supplement: Supplementary file 1 [file children-13-00586-s001.zip › children-4228340-supplementary.pdf]

Supplementary materials

S1: survey questionnaire

Survey Questionnaire

1. Which professional figures are present at your center? (Multiple choice)

- Child Neuropsychiatrist, Speech and Language Therapist (SLT), Neuropsychologist, Childhood Neuro- and Psychomotricity Therapist (TNPEE), Physiotherapist, Occupational Therapist.

2. How frequently do follow-up patients at your center undergo speech and language evaluations? (Multiple choice)

- Every 6 months, every 12 months, every 24 months, ad libitum.

3. Which patients primarily attend your center? (Multiple choice) GMFCS levels I-II-III; GMFCS levels IV-V [31];

4. Which tests, available at your facility, do you consider most suitable for assessing lexical comprehension across different age groups? (Multiple choice)

- MacArthur-Bates CDI-italian version, il Primo Vocabolario del Bambino-PVB [41]; Test di Primo Linguaggio – TPL [43]; *Parole in Gioco*- PinG [42]; New Reynell Developmental Language Scales-NRDLS [89]; Test Fono-lessicale-TFL [44]; Batteria per la valutazione del linguaggio- BVL– Lexical Comprehension [45]; Other.

5. Which tests do you consider most suitable for assessing morpho-syntactic comprehension? (Multiple choice)

- Prove per la comprensione verbale nella prima infanzia- COVER [50]; Test di Primo Linguaggio - TPL [43]; Prova di comprensione grammaticale con oggetti-PCGO [51]; BVL– Grammatical Comprehension [45].

6. Which tests do you consider most suitable for assessing lexical production? (Multiple choice)

- PVB [41]; TPL [43]; TFL [44]; NRDLS [89]; BVL – Lexical Production [45]; Other.

7. Which tests do you consider most suitable for assessing morpho-syntactic production? (Multiple choice)

- PVB [41]; Test di Primo Linguaggio – TPL [43]; NRDLS [89]; BVL– Sentence Repetition [45]; Sentence Repetition Test [55].

8. Which tests do you consider most suitable for assessing phonetic-phonological skills? (Multiple choice)

- Consonant inventory via spontaneous speech sampling; Prove per la valutazione fonologica del bambino- PFLI [77]; BVL– Naming and Articulation[45].

9. How do you assess speech intelligibility? (Open-ended)

10. How do you assess articulatory diadochokinesis? (Open-ended)

11. How do you assess speech variability? (Open-ended)

12. Which tools do you use to evaluate communication and pragmatics?

- Focus-I [60]; Other.

13. Do you have experience with Augmentative and Alternative Communication (AAC)? Which tools do you consider most suitable for determining the indication for AAC use? (Multiple choice)

- CAA Assessment [63]; ComFor-2 [64]; Other.

14. Which tests do you consider most suitable for assessing cognitive skills? (Multiple choice)

- Griffiths' III [32], WPPSI-IV [34], WISC-IV [36]; Other.

15. Which tests do you consider most suitable for assessing visual-perceptual skills? (Multiple choice)

- Test of Visual Perception-TPV [39]; Test of Visual-Motor Integration-VMI [41].

16. Would you be interested in participating in a study focusing on (Multiple choice):

- Assessment of children <36 months; Assessment of children aged 5–7 years; Assessment of minimally verbal children.

S2: protocols

S.11 Protocol for children <4 years

**LEXICON**	COMPREHENSION	Primo Vocabolario del Bambino - PVB [41],<br>Italian version of the "MacArthur-Bates-Communicative Development Inventory"<br>Parole in Gioco - PinG [42]<br>Test Fono-Lessicale - TFL [44]*
	PRODUCTION	PVB [41]<br>PinG [42]<br>TFL [44]
**SYNTAX**	COMPREHENSION	Prove di Valutazione della Comprensione Linguistica- PVCL [52]*
	PRODUCTION	PVB [41]<br>Test di valutazione del linguaggio - TVL [48]*
**SPEECH**	ARTICULATION	Consonant inventory (spontaneous speech sample)*
	INTELLEGIBILITY	The Intelligibility in Context Scale: Italian - ICS-I [58]*<br>Viking Speech Scale - VSS [84]
	LEXICAL VARIABILITY & DIADOCOKINESIS	Robbins & Klee italian adaptation [59]*
	PHONATORY FUNCTION	Robbins & Klee italian adaptation [59]*
**PRAGMATICS**		PVB [41]<br>Communication and Symbolic Behavior Scales - CSBS [23]<br>Italian Focus on the Outcomes of Communication Under Six - FOCUS-I [60]*

*to be used for children >3 years.

Table S1.2 Protocol for verbal children 4-12 years

**LEXICON**	COMPREHENSION	Batteria di Valutazione del Linguaggio - BVL [45]
	PRODUCTION	BVL [45]
**SYNTAX**	COMPREHENSION	BVL [45]
	PRODUCTION	BVL [45]
**SPEECH**	ARTICULATION	Consonant inventory (spontaneous speech sample)
	INTELLEGIBILITY	ICS-[58]<br>VSS [27]
	LEXICAL VARIABILITY & DIADOCOKINESIS	Robbins & Klee italian adaptation [59]
	PHONATORY FUNCTION	Robbins & Klee italian adaptation [59]
**PRAGMATICS**		FOCUS-I [60]

Table S1.3 Protocol for minimally verbal or nonverbal children 6-12 years

**LEXICON**	COMPREHENSION	BVL Comprensione Lessicale in età prescolare e Comprensione Lessicale in età scolare [45]
	PRODUCTION	PVB [41]
**SYNTAX**	COMPREHENSION	BVL [45] Comprensione Grammaticale
**PRAGMATICS**		CSBS [23]<br>Interview for the assessment of augmentative and alternative communication
